# The Feeding Practices and Structure Questionnaire: development and validation of age appropriate versions for infants and toddlers

**DOI:** 10.1186/s12966-021-01079-x

**Published:** 2021-01-19

**Authors:** E. Jansen, C. G. Russell, J. Appleton, R. Byrne, L. A. Daniels, C. Fowler, C. Rossiter, K. M. Mallan

**Affiliations:** 1 School of Exercise and Nutrition Sciences, Queensland University of Technology, Victoria Park Rd, Kelvin Grove, QLD 4059 Australia; 2 Division of Child & Adolescent Psychiatry, Department of Psychiatry & Behavioral Sciences, Johns Hopkins University School of Medicine, Baltimore, MD 21287 USA; 3 CASS Food Research Centre, Faculty of Health, School of Exercise and Nutrition Sciences, Deakin University, 1 Gheringhap St, Geelong, VIC 3220 Australia; 4 Susan Wakil School of Nursing and Midwifery, University of Sydney, 88 Mallett St, Camperdown, NSW 2050 Australia; 5 Tresillian Family Care Centres, McKenzie Street, Belmore, Sydney, NSW 2192 Australia; 6 Institute of Health and Biomedical Innovation, Centre for Children’s Health Research, Queensland University of Technology, 62 Graham St, South Brisbane, QLD 4101 Australia; 7 School of Nursing and Midwifery, Faculty of Health, University of Technology Sydney, 15 Broadway, Ultimo, NSW 2007 Australia; 8 School of Behavioural and Health Sciences, Australian Catholic University, 1100 Nudgee Rd, Banyo, QLD 4014 Australia

**Keywords:** Feeding practices, Structured mealtimes, Responsive feeding, Infants, Toddlers, Questionnaire, Development, Validation

## Abstract

**Background:**

In order to measure and understand trajectories of parental feeding practices and their relationship with child eating and weight, it is desirable to perform assessment from infancy and across time, in age-appropriate ways. While many feeding practices questionnaires exist, none is presently available that enables tracking of feeding practices from infancy through childhood. The aim of the study was to develop a version of the Feeding Practices and Structure Questionnaire (FPSQ) for parents with infants and toddlers (< 2 years) to be used in conjunction with the original FPSQ for older children (≥2 years) to measure feeding practices related to non-responsiveness and structure across childhood.

**Methods:**

Constructs and items for the FPSQ for infants and toddlers were derived from the existing and validated FPSQ for older children and supplemented by a review of the literature on infant feeding questionnaires. Following expert review, two versions of the questionnaire were developed, one for milk feeding parents and one for solid feeding parents. Data from two studies were combined (child ages 0–24 months) to test the derived constructs with Confirmatory Factor Analysis for the milk feeding (*N* = 731) and solid feeding (*N* = 611) versions.

**Results:**

The milk feeding version consisted of four factors (18 items) and showed acceptable model fit and good internal reliability: ‘feeding on demand vs. feeding routine’ (α = 0.87), ‘using food to calm’ (α = 0.87), ‘persuasive feeding’ (α = 0.71), ‘parent-led feeding’ (α = 0.79). The same four factors showed acceptable model fit for the solid feeding version (21 items), likewise with good internal reliability (α = 0.74, 0.86, 0.85, 0.84 respectively). Two additional factors (13 items) were developed for the solid feeding version that appeared developmentally appropriate only for children aged 12 months or older: ‘family meal environment’ (α = 0.81) and ‘using (non-)food rewards’ (α = 0.92). The majority of factor-factor correlations were in line with those of the original FPSQ.

**Conclusions:**

The FPSQ milk and solid feeding versions are the first measures specifically developed as precursors to the FPSQ to measure parental feeding practices in children < 2 years, particularly practices related to non-responsiveness and structure. Further validation in more diverse samples is required.

**Supplementary Information:**

The online version contains supplementary material available at 10.1186/s12966-021-01079-x.

## Background

The number of children under 5 years of age with overweight is projected to rise from 40 million to 43 million by 2025 [1–3]. Increases have been observed in the prevalence of childhood overweight in every continent, and across high, low- and middle-income countries [3]. Although there is evidence of plateauing in some countries, this is at high levels [1]. UNICEF noted recently that “strikingly, there is little or no consistent evidence of countries achieving and sustaining a decline in obesity across the population since the 1980s, underlying the need to focus on prevention.” ( [3] p.48). Underscoring the importance of designing effective prevention efforts is evidence that both weight loss and maintenance after weight loss are difficult, and children who gain excess weight are therefore likely to continue to live with overweight in adulthood [4]. Accordingly, research efforts are increasingly being directed towards understanding how and why obesogenic eating behaviours develop in some individuals early in life.

Although parental feeding practices have been identified as important in explaining differences in the development of children’s eating behaviours and weight, findings from longitudinal studies are mixed. This can partly be explained by differences in study design and measurement: parent-child interactions and outcomes are typically examined over relatively short (< 1–3 years) periods, beginning at different ages across childhood, and utilising a range of measurement tools [5–12]. Very few studies (The Generation R study is an exception [13]), have begun examination of parent-child feeding interactions and outcomes in infancy and have continued this analysis into childhood or adolescence. Consequently, many questions remain unanswered in relation to bidirectional and transactional influence processes occurring between parents and infants/children in relation to eating and growth outcomes across various ages and stages of childhood. Studies utilising longitudinal designs that begin in infancy and extend into middle childhood and beyond would provide knowledge about the developmental trajectories and processes that are causally linked to eating and weight outcomes that could inform prevention efforts [14].

To date, studies of feeding practices with infants have been limited by a lack of appropriate measurement tools. Across the body of work on parental feeding, there have been several approaches to measurement. Examples of questionnaires include the Infant Feeding Questionnaire (IFQ) [15], Infant Feeding Style Questionnaire (IFSQ) [16], or Lakshman et al.’s questionnaire on maternal attitudes towards infant growth and milk feeding practices (LMFQ) [17]. Research and assessment have frequently focused on controlling feeding practices, although a wide range of feeding practices has been identified, including structure and autonomy support, and these have been linked to outcomes in early childhood [18–20]. Yet inconsistent and non-comprehensive measurement tools have limited our understanding of the role and influence of parental feeding practices in a developmental way [21]. This is exacerbated by the absence of a tool that can measure appropriate feeding practices constructs across both infancy and childhood (i.e. over the continuum of child development) or throughout longitudinal studies. The development of a parental feeding practices tool appropriate for infancy, that measures the same or similar constructs to those in older age groups (2 years plus) would enable researchers to understand processes underlying associations between parent feeding and child eating and weight prospectively. It would also facilitate exploration of how parental feeding practices change over time for different parents and children, as well as between different feeding modes (breastfeeding, bottle feeding, solid and family foods), and the impact of practices upon eating and weight outcomes.

In developing the Feeding Practices and Structure Questionnaire (FPSQ), Jansen et al. [22] attempted to address some of the abovementioned issues, providing a theoretically-driven and conceptually coherent measure of two authoritative feeding practices domains associated with children’s self-regulation of food intakes and healthy eating: i. parental feeding responsiveness to children’s hunger and satiety cues, and ii. mealtime structure [22]. Within the original FPSQ development sample, acceptable concurrent validity, construct validity and internal reliability of the FPSQ were reported [22, 23]. The authors subsequently examined longitudinal measurement invariance and reduced the number of items from 40 to 28 (FPSQ-28) [24], providing evidence that the FPSQ-28 is appropriate for use in longitudinal studies of children aged 2–5 years of age. To facilitate longitudinal studies with multiple measurement points across childhood, there is a need to develop a comparable tool to measure authoritative feeding practice domains in infancy. Information obtained from a FPSQ for infants (< 2 years) would not only enable the design of more effective obesity prevention efforts that could begin early in life and are tailored to the unique challenges of particular parent-child dyads, but, in partnership with the existing FPSQ also enable assessment of authoritative feeding practices domains throughout intervention studies that cross several child-developmental stages.

The aims of the present paper were therefore to modify the FPSQ for infants and toddlers (aged < 2 years) and test the factorial validity of two age-appropriate versions of the FPSQ, one for infants who are predominantly milk fed and one for infants who are predominantly fed solid foods. Given that authoritative feeding constructs and their measurement may differ in infants from toddlers and older children, the original, larger, 40 item FPSQ was taken as the base for development.

## Methods

### Participants and procedures

**Fig. 1:**
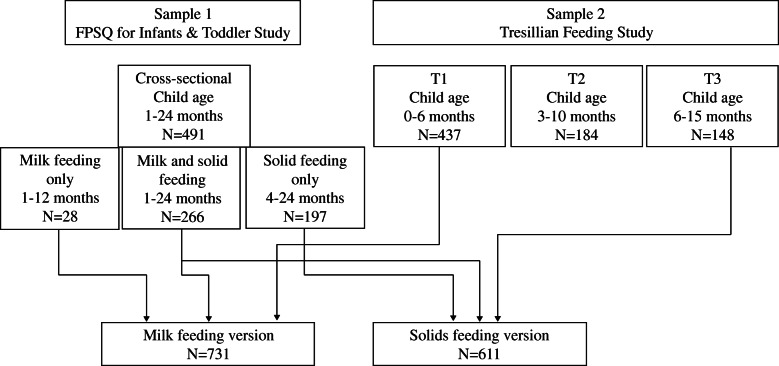
Overview of the samples utilised for the development and validation of the milk (FPSQ-M) and solids (FPSQ-S) feeding questionnaire versions including assessment time point (Sample 2 only), child age range, and sample size. Note: T2 of Sample 2 was not utilised in the present study in order to not include the same participants twice in the development of the FPSQ-M

Data for this study came from two Australian research projects both of which had the development and validation of the Feeding Practices and Structure Questionnaire for infants and toddlers as primary (Sample 1) or secondary aim (Sample 2) (see Fig. 1).

*Sample 1 (“the FPSQ for Infants and Toddlers Study”)* was specifically designed to develop, test and validate the Feeding Practices and Structure Questionnaire for infants and toddlers. Sample 1 was recruited as a supplement to Sample 2, running concurrently with the follow-up assessments of Sample 2, to specifically cover the child age range 12–24 months and expand some of the feeding practices concepts that were not necessarily age appropriate in under 12-month-olds and therefore not examined in Sample 2 (e.g. using rewards). Parents were recruited through university staff and student email distribution lists, parenting forums and social media websites that were exclusively targeted towards Australian parents. For instance, social media websites included Australian Facebook groups (e.g. mums and bubs groups; location specific pregnancy, baby and child buy/swap/sell groups). Eligible participants were 18 years or above, had internet access to the online survey; their child was between 6 and 24 months old and had not been diagnosed with any feeding disorder. In total, 530 participants commenced the online survey. Of those, 491 provided relevant data for the development and validation of the FPSQ for infants. Notably, 19 responses from parents with children younger than 6 months (minimum age 1 month) were accepted due to overlap with the same age of Sample 2.

Respondents completed the self-administered online questionnaire and reported on the following demographic variables: child age, child gender, parent age, parent gender, relationship with child, feeding responsibility, feeding mode and education level. Additionally, participants indicated whether or not they were of Aboriginal or Torres Strait Islander origin (0.8% yes). Participants could go into the draw to win one of four AUD25 gift vouchers. Approval was obtained from the Queensland University of Technology Human Research Ethics Committee (REF NO. 1400000033).

*Sample 2* (*the Tresillian Feeding Study*) was designed to examine the feeding practices of Australian parents of infants and how practices related to the development of infant eating and weight longitudinally. Participants were recruited at the Tresillian Family Care Centres (https://www.tresillian.org.au/), an early parenting support service, in the state of New South Wales, Australia and via advertisements posted to the Tresillian Facebook group. At the Tresillian Family Care Centres (residential and day stay centres), flyers and posters were displayed around the centres and handed to parents or caregivers by Tresillian nurses. Interested parents and caregivers were provided with a plain language information sheet prior to consenting to participate. They returned their completed paper-and-pencil questionnaire to a sealed box for subsequent collation and data entry by research staff. Parents and caregivers who responded to the flyer posted on the Tresillian Facebook group were directly linked to an online version of the plain language information sheet and survey, hosted on SurveyGizmo. To be eligible for participating in the survey, parents/caregivers needed to be 18 years or older, have an infant less than 6 months of age, and be able to read and write in English. Participants could enter into a draw to win one of two iPads. In total 496 participants provided some data. Participants were excluded if their baby was older than 6 months of age at baseline, less than 35 weeks gestation, < 2500 g birthweight, living outside Australia, had a health condition that affected feeding, or if data on feeding practices were missing. In total, 59 participants were excluded, leaving 437 participants with relevant data for the development and validation of the FPSQ for infants at baseline assessment (child ages 0–6 months) and 148 participants at follow-up assessment (child ages 6–15 months).

Respondents completed the self-administered (online) questionnaire which included the following demographic variables: child age, gender, multiple birth, parent age, gender, relationship with child, feeding responsibility, feeding mode and education level. Participants of Sample 2 also indicated their country of birth (86% were born in Australia) and whether or not their child is of Aboriginal or Torres Strait Islander origin (4.3% yes). Ethical approval was granted by the Sydney Local Health District Human Research Ethics Committee (Protocol No X15–0233) and the University of Technology Sydney Human Research Ethics Committee (REF NO. 2015000528).

### Generation of constructs and items

**Fig. 2:**
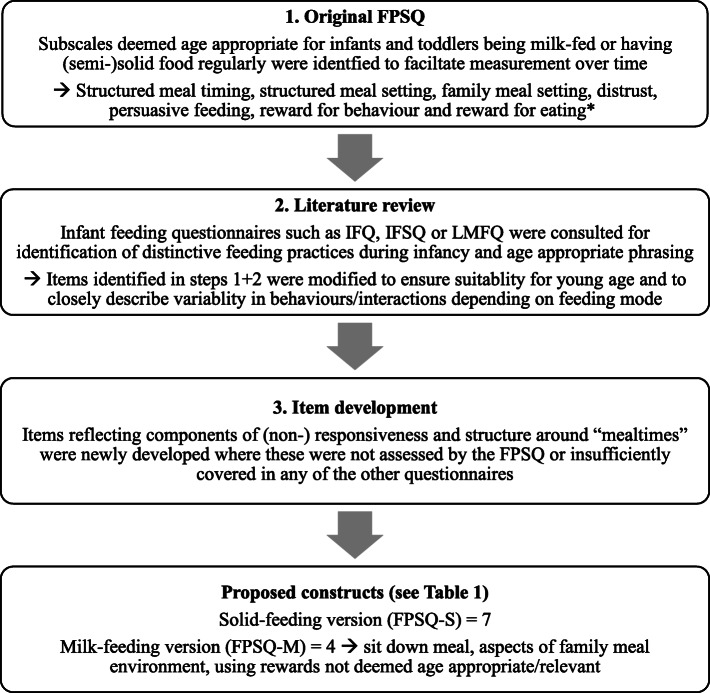
Flowchart of construct and item generation. Abbreviations: FPSQ = Feeding Practices and Structure Questionnaire [22], IFQ = Infant Feeding Questionnaire [15], IFSQ = Infant Feeding Style Questionnaire [16], LMFQ = Lakshman et al.’s questionnaire on maternal attitudes towards infant growth and milk feeding practices [17]. * Overt and covert restriction were not considered age appropriate and since further conceptual understanding [25] is needed to clarify mixed findings in relation to the restrictive feeding construct [26], it was left out of the current FPSQ version for infants and toddlers

Generation of constructs and items was based initially on the original FPSQ and its underlying theory of authoritative feeding [22], and were adapted for use with children under the age of 2 years. Thus, item construction and selection were mainly conducted a priori. Due to the differences in developmental stages of infants and the feeding mode (milk, or [semi-] solid foods), it was necessary to devise two versions: one for milk feeding interactions (FPSQ-M) and one for (semi-)solid feeding interactions (FPSQ-S). The feeding practices constructs and items to be included in the infant and toddler version of the questionnaire were derived in three ways (see Fig. 2). Proposed constructs are shown in Table 1.

**Table 1:** Overview of proposed feeding constructs for the milk (FPSQ-M) and solid-feeding (FPSQ-S) version of the FPSQ, matched with the original FPSQ constructs

Component	FPSQ-M	FPSQ-S	FPSQ – original [10]	
Mealtime structure (environment)	Feeding routine vs. feeding on demand	Feeding routine vs. feeding on demand	Structured Meal Timing	Structure
	NA Due to age/dev. Stage	Feeding location – sit down meal	Structured Meal Setting	
	NA Due to age/dev. Stage	Family meal environment	Family Meal Setting	
Feeding initiation & termination (duration, how much) – (non-) reliance on cues	Parent-led feeding	Parent-led feeding	Distrust	Non-responsiveness
	Persuasive Feeding	Persuasive Feeding	Persuasive Feeding	
Feeding for reasons of hunger or other reasons	Food to calm	Food to calm	Reward for Behaviour	
	NA Due to age/dev. Stage	Using (non-)food rewards	Reward for Eating	

In the next phase, proposed constructs for the milk and solid feeding versions respectively were independently reviewed by 12 international experts, chosen based on their clinical and/or research experience in infant feeding. The experts’ feedback confirmed the decision to develop two versions of the feeding practices questionnaire based on the feeding mode (i.e. infant still having milk feeds vs. infant having (semi-)solid feeds, depending on how the parent would currently mainly feed their child), rather than splitting the questionnaire by age (e.g. < 6 months, 6–12 months and > 12 months). Based on feedback to increase the distinction between both questionnaire versions, terminology was adjusted so that the milk feeding version refers to ‘baby’ and ‘feeding’ (e.g. “I let my baby decide when he would like to have a feed”), while the solid feeding version refers to ‘child’ and ‘eating’ (e.g. “I let my child decide when he would like to eat”). Finally, the revised questionnaires were piloted with 4 participants. While most comments related to the length and ease of online completion, another suggestion included providing a time frame (e.g. think about feeding your baby within the last two/few weeks) as feeding interactions at this early age change quickly. Inclusion of a ‘not applicable’ option was also suggested due to variability in developmental stages. All items were scored on a 5-point Likert scale with responses from 1 = never to 5 = always. A ‘not applicable’ response option was available for all items.

### Item consolidation, factor identification, specification and validation

Once data were collected for both questionnaire versions in the Samples 1 and 2, items were closely screened. Issues with response distribution and normality (i.e. kurtosis values > 3 and high skewness as visually inspected via histograms) were noted down for each item. Similar to Llewellyn et al. [27], items were discarded if > 80% of respondents ticked the same response option (e.g. ‘never’ or ‘always’) or if a large number of respondents (> 5%) selected the ‘not applicable’ response option. Next, Samples 1 and 2 were combined to conduct Confirmatory Factor Analysis for both versions of the questionnaire (see Fig. 1).

### Confirmatory factor analysis

Confirmatory Factor Analysis (CFA) was conducted for the statistical construct specification of the full model. The aim was not only to verify the proposed model, but also to identify the strongest set of items for each feeding construct, confirm the factorial validity, flag issues with cross-loading items, and examine factor-factor correlations. Furthermore, CFA was chosen in order to follow the development procedure for the original FPSQ questionnaire. Given the large sample size and items being developed for particular constructs, thus providing a specific model to be tested against the observations, CFA was selected as preferred method. CFAs were conducted in Mplus Version 7.3 [28] with the weighted least squares estimator (WLSMV; for ordinal categorical indicators) [29]. Model specifications included fixing one regression weight per factor to 1 and correlating all factors with one another. The following indices and acceptable cut-offs were used to evaluate overall model fit: the normed chi-square (χ2/df) with values between 1.0–2.0, Comparative Fit Index (CFI) and Tucker-Lewis Index (TLI) > 0.90, and Root Mean-Square Error of Approximation (RMSEA) < 0.08 [30–32]. Post hoc modifications were undertaken to improve model fit if acceptable levels were not achieved. Model re-specifications, such as loading an item onto another subscale than initially designed, were guided by modification indices and conceptual justifications. Items identified as having poor measurement properties (i.e. non-significance with *p* ≥ 0.001, item-factor loading < 0.4, squared multiple correlation < 0.2) were removed. Internal consistency was determined using Cronbach’s alpha and Coefficient *H*. As for the original development of the FPSQ, subscales with values < 0.6 were deemed to have poor reliability and consequently excluded [22].

## Results

Sample characteristics of Samples 1 and 2 with relevant feeding practices data are presented in Table 2. Characteristics are presented for the four subsamples that were used for the different analysis steps. Notably, 266 participants of Sample 1 were currently milk and solid feeding their child and therefore completed both sets of questions. Consequently, 294 participants from Sample 1 and 437 from Sample 2 (T1 assessment) contributed to the milk feeding version (*N* = 731), while 463 participants from Sample 1 and 148 from Sample 2 (T3 assessment) contributed to the solids feeding version (*N* = 611).

**Table 2:** Sample characteristics of participants from samples 1 and 2 by FPSQ version

		Milk feeding version *N* = 731		(Semi-) Solid feeding version *N* = 611	
		Sample 1 *n* = 294^a^	Sample 2 *n* = 437^a^	Sample 1 *n* = 463^a^	Sample 2 *n* = 148^a^
Child gender	Girl	136 (46.6%)	199 (46.4%)	217 (47.2%)	57 (41.0%)
Child age in months	M ± SD	11.71 ± 5.28	2.61 ± 1.50	14.28 ± 5.69	10.15 ± 1.71
Multiple birth	Twin	1 (0.3%)	1 (0.2%)	6 (1.3%)	0
	Triplet or higher	0	0	1 (0.2%)	0
Feeding mode	Currently BF	212 (72.1%)	358 (82.5%)	200 (50.0%)	87 (58.8%)
	Weaned	73 (24.8%)	67 (15.4%)	181 (45.3%)	59 (39.9%)
	Never BF	9 (3.1%)	9 (2.1%)	19 (4.8%)	2 (1.4%)
	Still has FF	108 (37.4%)	157 (35.9%)	105 (26.5%)	85 (57.4%)
	Previously FF	15 (5.2%)	59 (13.5%)	97 (24.5%)	14 (9.5%)
	Never FF	171 (58.2%)	221 (50.6%)	194 (49.0%)	49 (33.1%)
	Not yet solids	28 (9.6%)	338 (77.3%)	0	0
	Yes solids	266 (90.8%)	99 (22.7%)	463 (100%)	148 (100%)
Relationship to child	Mother	279 (95.2%)	389 (99.7%)	440 (95.2%)	138 (100%)
	Father	14 (4.8%)	1 (0.3)	21 (4.5%)	0
	Relative	0	0	1 (0.2%)	0
Feeding responsibility	Mostly/entirely me	241 (82.0%)	366 (94.8%)	360 (77.8%)	126 (86.3%)
Parent age	M ± SD	32.34 ± 5.91		32.50 ± 5.76	
	< 20	1 (0.3%)	1 (0.3%)	2 (0.4%)	0
	20–29	79 (27.0%)	154 (39.5%)	114 (24.7%)	52 (37.7%)
	30–39	196 (66.9%)	228 (58.4%)	311 (67.6%)	84 (60.9%)
	40+	17 (5.8%)	7 (1.8%)	33 (7.2%)	2 (1.4%)
Highest education level	Uni or higher	186 (63.3%)	225 (58.7%)	294 (63.5%)	98 (71.0%)

^a^Sample sizes vary due to skipped responses or missing data. Abbreviations: *BF* breastfeeding, *FF* formula feeding

### Development and validation of the FPSQ milk feeding version – FPSQ-M

#### CFA – factor specification and structural validation (*N* = 731)

Initially 26 items were developed for the milk-feeding version of the FPSQ. Six items were excluded based on serious issues with item distribution (all from the Parent-led feeding construct; see Additional file 1 for table with excluded items). The remaining 20 items for which less severe issues with item distribution were flagged were included in the CFA. The proposed model, based on the theoretical development of the FPSQ milk-feeding version, consisted of four factors: ‘Feeding on demand’ (4 items), ‘Using food to calm’ (5 items), ‘Persuasive feeding’ (5 items) and ‘Parent-led feeding’ (6 items). This initial model showed poor fit (RMSEA = 0.110, CFI = 0.90 and TLI = 0.89) and two items loaded < 0.4 onto the Persuasive Feeding factor (PERS 3 + 4).

Modification indices indicated that PERS3 (“I offer the breast/bottle to check if my baby is hungry”) might better load with the factor ‘Using food to calm’. While model fit improved (RMSEA = 0.075, CFI = 0.96 and TLI = 0.95), PERS4 still loaded below < 0.4 and was removed in the next step. Goodness-of-fit indices indicated that this model showed acceptable fit to the data (RMSEA = 0.080, CFI = 0.95 and TLI = 0.95). However, after examination of all items on the factor it was decided that this item did not fully fit with the remaining items. Removal of PERS3 led to a model of mostly acceptable fit (RMSEA = 0.082, CFI = 0.96 and TLI = 0.95) while the normed chi-square of 5.9 was outside the desirable range. All items were significant, had standardised factor loadings > 0.5 and SMC values ≥0.2. Cronbach’s alphas, Coefficient *H*, mean scores and factor-factor correlations are presented in Table 4, reflecting good internal reliabilities with all Cronbach’s alphas being above 0.7 and all Coefficient *H*s being above 0.8 (Tables 3 and 4).

**Table 3:** Standardised factor loadings for items of the FPSQ-M according to CFA (*N* = 731) – 4 factors, 18 items

Factor	Label	Item	Loading
Feeding on demand	DEM1	I feed my baby whenever he wants	.862
	DEM2	I feed my baby at set times^a^	.840
	DEM3	I decide when it is time for my baby to have a feed^a^	.803
	DEM4	I let my baby decide when he would like to have a feed	.921
Using food to calm	FC1	I feed my baby to settle him, even if he is not hungry	.785
	FC2	I offer my baby a feed when he is unsettled or crying	.885
	FC3	I offer my baby a feed when he is hurt	.838
	FC4	When my baby gets unsettled or is crying, feeding him is one of the first things I do	.851
	FC5	I feed my baby to make sure that he does not get unsettled or cry	.678
Persuasive feeding	PERS1	I feed my baby extra milk, just to make sure he gets enough	.761
	PERS2	If my baby indicates he is not hungry, I try to get him to feed anyway	.656
	PERS5	I feed my baby extra milk so he sleeps longer	.832
Parent-led feeding	PARENT1	When deciding how much to feed my baby, I rely on how hungry he is^a^	.582
	PARENT 3	I feed my baby for a set time	.573
	PARENT 5	I carefully control how much my baby feeds	.871
	PARENT 6	I follow a rule about how much my baby should feed	.848
	PARENT 7	I let my baby decide how much he feeds^a^	.811
	PARENT 8	I decide how much my baby feeds	.785

^a^Item is reverse coded

Response options: 1 = never, 2 = rarely, 3 = sometimes, 4 = often, 5 = always

**Table 4:** Internal reliability, means, standard deviation and factor-factor correlations of 4 milk-feeding practices (based on 18 items, FPSQ-M) – *N* = 731

	Demand 4 items	Food to calm 5 items	Persuasive feeding 3 items	Parent-led feeding 6 items
Cronbach’s alpha	.868	.874	.713	.789
Coefficient H	.927	.917	.814	.911
Mean ± SD	3.77 ± 1.01 2 missing	2.57 ± 0.93 16 missing	1.91 ± 0.80 16 missing	1.67 ± 0.75 0 missing
Food to calm	0.490 (<.001)	1		
Persuasive feeding	−0.259 (<.001)	0.277 (<.001)	1	
Parent-led feeding	−0.646 (<.001)	−0.309 (<.001)	0.426 (<.001)	1

### Development and validation of the solid feeding version – FPSQ-S

#### CFA – factor specification and structural validation (*N* = 611)

Initially 35 items were developed for the solid-feeding version of the FPSQ. The same six items as above were excluded based on serious issues with item distribution (all from the Parent-led feeding construct; see Additional file 1 for table with excluded items). The remaining 29 items for which less severe issues with item distribution were flagged were included in the CFA. The proposed model, based on the theoretical development of the FPSQ solid-feeding version and the established factors of the milk-feeding version, consisted of four factors: ‘Feeding on demand’ (5 items), ‘Using food to calm’ (6 items), ‘Persuasive feeding’ (11 items) and ‘Parent-led feeding’ (7 items). This initial model showed poor fit (RMSEA = 0.076, CFI = 0.89 and TLI = 0.88) and four items had factor loadings < 0.4 (PARENT2, PERS 5 + 6 + 11). Additionally, modification indices revealed that several items might better load onto a different factor than currently placed. Consequently, several changes were made to the model in subsequent iterations. These included: 1) moving PERS5 to the Parent-led feeding factor but then removing it in the next step because of a standardised factor loading < 0.4; 2) moving PERS11 to the Parent-led feeding factor but then removing it in the next step because of a standardised factor loading < 0.4; deleting 3) PARENT2 and 4) PERS6 because of standardised factor loadings < 0.4, 5) PARENT1 because of content-overlap with PERS9 and cross-loading with the Persuasive feeding factor, and finally 6) DEM1, PARENT3 and PERS3 because of parsimony (i.e. better model fit was achieved with fewer items making them redundant; see Additional file 1 for model fit). The final model included 21 items, loading onto 4 factors. Goodness-of-fit indices indicated that this model showed acceptable fit to the data: RMSEA = 0.065, CFI = 0.95 and TLI = 0.94, while the normed chi-square of 3.6 was outside the desirable range. All items were significant, had standardised factor loadings > 0.5 (see Table 5) and SMC values > 0.2.

**Table 5:** Standardised factor loadings for items of the FPSQ-S according to CFA (4 factors, 21 items and for the extended version – 6 factors 34 items)

Factor	Label	Item	Loading ***N*** = 611	Loading extended version ***N*** = 463
Feeding on demand (lower score indicates feeding on demand)	DEM2	My child eats at set times	.850	.825
	DEM3	I decide when it is time for my child to eat	.632	.648
	DEM4	I let my child decide when she/he would like to eat^a^	.628	.630
	DEM5	My child has a set mealtime routine	.786	.789
Using food to calm	FC1	I give my child food to settle him/her even if he/she is not hungry	.698	.731
	FC2	I offer my child something to eat to make her/him feel better when she/he is unsettled or crying	.882	.872
	FC3	I offer my child something to eat to make her/him feel better when she/he is hurt	.863	.934
	FC4	When my child gets unsettled or is crying, one of the first things I do is give her/him food	.776	.793
	FC5	I give my child food to make sure that they do not get unsettled or cry	.716	.704
	FC6	I use food to distract my child or keep him/her busy	.641	.650
Persuasive feeding	PERS1	I encourage my child to eat all of the food in front of him/her	.795	.803
	PERS2	When my child turns away, I try to get her/him to eat a little bit more	.800	.774
	PERS4	If my child indicates she/he is not hungry I try to get her/him to eat anyway	.719	.737
	PERS7	I say or do something to show my disapproval of my child for not eating	.682	.761
	PERS8	I praise my child after each bit to encourage finishing the food	.682	.710
	PERS9	When my child refuses food they usually eat, I encourage her/him to eat it	.708	.714
	PERS10	I play games to make sure my child eats enough	.645	.681
Parent-led feeding	PARENT6	I carefully control how much my child eats	.800	.808
	PARENT 7	I have a rule about how much my child should eat	.851	.853
	PARENT 8	I let my child decide how much she/he eats^a^	.756	.793
	PARENT 9	I decide how much my child eats	.767	.811
Family Meal Environment	FM3	My child eats together with other family members.	X	.816
	FM4	My child is given the same foods as the rest of the family (pureed, mashed, chopped).	X	.788
	FM5	Whether my child is eating or not, my child sits with the rest of the family when they are having a meal.	X	.611
	FM6	I eat my meals while my child eats.	X	.864
Using (non-) food rewards	REW1	I offer foods to my child as a reward for good behaviour.	X	.859
	REW2	I offer my child their favourite foods in exchange for good behaviour.	X	.913
	REW5	I promise my child something other than food if they eat (for example: “If you eat your beans, we can go to the park”).	X	.861
	REW6	When my child refuses food they usually eat, I encourage eating by offering a non-food reward (for example: favourite toy or sticker).	X	.884
	REW7	I encourage my child to eat something by using food as a reward (for example: “If you finish your vegetables, you will get some dessert”).	X	.960
	REW8	When my child refuses food they usually eat, I encourage eating by offering a food reward (for example: dessert).	X	.918
	REW9	I use desserts as an encouragement to get my child to eat the main course.	X	.960
	REW10	I make my child finish the main course before having a dessert.	X	.703
	REW11	I warn my child that I will take a favourite food away if my child does not eat a food they do not like (for example: “If you don’t finish your vegetables, you won’t get dessert”).	X	.870

^a^Item is reverse coded

X item was not tested in this version

Response options: 1 = never, 2 = rarely, 3 = sometimes, 4 = often, 5 = always

As shown in Table 1, three additional feeding constructs were included in the solid-feeding version of the FPSQ compared to the milk-feeding version [i.e. (1) “Feeding location – sit down meal”, (2) “Family meal environment” and (3) “Using (non-)food rewards”]. Due to the younger age and thus different developmental stage of the children in Sample 2, these three feeding aspects were only investigated in Sample 1 (*n* = 463). Initially 10 and 11 items were developed to assess mealtime structure (constructs 1 and 2) and using (non-)food rewards (construct 3). Four items were excluded based on serious issues with item distribution (2 from constructs 1 and 3 respectively; see Additional file 1 for table with excluded items). Issues with item distribution were flagged for the remaining 17 items.

Next, the final CFA model for the solid-feeding version presented above with 4 factors (21 items) was used and 2 new factors were added – mealtime structure (8 items) and rewards (9 items). Although the goodness-of-fit indices indicated that this model showed acceptable fit to the data (RMSEA = 0.066, CFI = 0.93 and TLI = 0.93, normed chi-square = 3.0), not all items were significant and modification indices revealed that several items might better load onto a different factor than currently placed. Additionally, standardised factor loadings around 0.4 suggested that some items should possibly be deleted. Consequently, six changes were made to the model in subsequent iterations. These included: first moving FME1 to the Rewards factor and then deleting it because the standardised factor loading was below 0.4; next moving FME2 to the Rewards factor and then deleting it because the standardised factor loading was below 0.4; finally deleting SIT3 and SIT2 due to standardised factor loadings below 0.4 (see Additional file 1 for model fit). The final model included 34 items, loading onto 6 factors. Goodness-of-fit indices indicated that this model showed acceptable fit to the data: RMSEA = 0.052, CFI = 0.97 and TLI = 0.96, while the normed chi-square of 2.2 was just outside the desirable range. All items were significant, had standardised factor loadings > 0.5 and SMC values ≥0.2. Cronbach’s alphas, Coefficient *H*, mean scores and factor-factor correlations are presented in Table 6, reflecting good internal reliabilities with all Cronbach’s alphas being above 0.7 and all Coefficient *H*s being above 0.8.

**Table 6:** Internal reliability, means, standard deviation and factor-factor correlations of 6 solid-feeding practices (based on 34 items, FPSQ-S; Sample 1, *n* = 463)

	Demand 4 items	Food to calm 6 items	Persuasive feeding 7 items	Parent-led feeding 4 items	Family meal environment 4 items	Using (non-) food rewards 9 items
Cronbach’s alpha	.740	.858	.853	.836	.805	.918
Coefficient H	.838	.936	.890	.891	.878	.979
Mean ± SD	3.33 ± 0.77 0 missing	1.80 ± 0.66 40 missing	2.64 ± 0.83 40 missing	2.06 ± 0.86 20 missing	3.74 ± 0.82 1 missing	1.56 ± 0.75 86 missing^a^
Food to calm	−0.135 (0.011)	1				
Persuasive feeding	0.359 (<.001)	0.361 (<.001)	1			
Parent-led feeding	0.440 (<.001)	0.218 (<.001)	0.613 (<.001)	1		
Family meal setting	−0.08 (0.110)	− 0.029 (0.603)	− 0.107 (0.040)	−0.248 (<.001)	1	
Using (non-) food rewards	0.096 (0.112)	0.560 (<.001)	0.590 (<.001)	0.396 (<.001)	0.129 (0.051)	1

^a^This is largely due to the “not applicable” response option, which was coded ‘missing’ for analysis

## Discussion

This paper reports on the development of two feeding practices questionnaires that align with the practices assessed in the FPSQ and can be used in infancy and toddlerhood. When used in conjunction with the FPSQ, the FPSQ-M and FSPQ-S are anticipated to allow for a more consistent method to measure and track key parental feeding practices from infancy into middle childhood. Following the same development and validation procedures as for the original FPSQ, two questionnaire versions were created – one for children currently being predominantly milk-fed (FPSQ-M), the other one for children currently being predominantly (semi-)solid-fed (FPSQ-S). The milk feeding version consisted of four feeding practices (feeding on demand vs. feeding routine, parent-led feeding, persuasive feeding and using food to calm) assessed with 18 items. The solid feeding version consisted of the same four feeding practices assessed with 21 items and additionally included two extra feeding practices that may only be relevant for children aged 12 months and older (family meal environment and using [non-]food rewards), which were assessed with 13 items. It is anticipated that researchers would use FPSQ-M for those infants predominantly milk-fed and the FSPQ-S for those predominantly fed solid foods. For example, in the study including Sample 2, in the follow-up assessment, parents were asked how they were mainly feeding their child and were directed based on their response to either the FPSQ-M or FPSQ-S items.

The current study revealed that four (for milk-fed infants) and six (for solid-fed infants) distinct and measurable feeding practice factors exist in children under the age of 2 years. As expected, given that the FPSQ-M and FPSQ-S were modelled on the FPSQ and its associated underlying theory, these largely aligned with the original FPSQ, as outlined in Table 1. ‘Feeding on demand vs. feeding routine’ (FPSQ-M: 4 items, FPSQ-S: 4 items) is hypothesised to align with the practice ‘structured meal timing’ and related to the parent making the decision *when* the child should feed (e.g. has set times, compared to letting the child decide). Notably, at this stage it is unclear if feeding on demand (whereby the infant decides the timing of feeding and has less of a routine) [33] is beneficial only up to a certain developmental point and beyond this point, structure and routine become more beneficial for the child (e.g. not grazing throughout the day but having set snack and mealtimes) [19]. Whether and how this relates to the child’s capability to self-regulate intake and the provision of healthy or unhealthy food needs further investigation. ‘Family meal environment’ (FPSQ-S: 4 items) is hypothesised to align with the factor ‘family meal setting’ and relates to the eating context, that is, are other family members present and is the child eating the same food as those other members. Items relating to the feeding location (e.g. a sit down meal at a table) that were hypothesised to be equivalent to the factor ‘structured meal setting’ did not form a distinct factor, but were removed throughout the model fitting process. This indicates either that for parents of younger children this feeding practice is not as relevant as it is for older children, or that the current items require improvement to better capture interactions related to the setup of the feeding environment that might support child self-regulation of eating. ‘Parent-led feeding’ (FPSQ-M: 6 items, FPSQ-S: 4 items) was hypothesised to align with the factor ‘distrust’ of the original FPSQ and relates to the parent making the decision (or having a rule) about *how long/how much* the child feeds. While this factor has been excluded from the more parsimonious FPSQ-28 due to a very strong correlation with the factor ‘persuasive feeding’, it was included in the infant and toddler version since decisions around feeding initiation and termination were revealed in the literature [34] and other questionnaires (e.g. 17) as important aspects of feeding at this age. ‘Persuasive feeding’ (FPSQ-M: 3 items, FPSQ-S: 7 items) was hypothesised to align with the factor ‘persuasive feeding’ and relates to the parent encouraging (pressuring) the child to eat more, even when showing signs of satiation. ‘Using food to calm’ (FPSQ-M: 5 items, FPSQ-S: 6 items) was hypothesised to align with the factor ‘rewards for behaviour’ and relates to the parent using food for settling or managing emotions. ‘Using (non-) food rewards’ (FPSQ-S: 9 items) was hypothesised to align with the factor ‘rewards for eating’ and relates to the parent using food to reward good behaviour or using (non-)food rewards to make the child eat unwanted food/finish their plate.

Model fit for both questionnaire versions was acceptable, although the solid feeding version was better, with lower RMSEA and normed chi-square values. This indicates that the model and thus the chosen questionnaire items showed a better fit to the data stemming from parents of the older children, possibly highlighting that feeding practices at this age can be more robustly measured and are more stable and therefore easier for parents to report on, while there is more complexity in capturing the actual feeding interaction that happens in infancy. Nonetheless, internal consistency of all feeding aspects assessed across the two versions was good with the lowest Cronbach’s alpha values for persuasive feeding (FPSQ-M: 0.71) and feeding on demand vs. feeding routine (FPSQ-S: 0.74) respectively.

Similar to the FPSQ-28, several items were seen in the current two versions that showed very high standardised factor-loadings. These may indicate that these items by themselves are sufficient to capture the constructs of interest. Using these single-item indicators instead of multi-item scales would reduce the length of the parent-reported questionnaire which in turn could reduce participant burden. In the FPSQ-M, one item of the feeding on demand vs. feeding routine construct had a standardised factor loading of 0.92 (“I let my baby decide when he would like to have a feed”). The loadings of the other three items ranged from 0.80 to 0.86, also showing high loadings. Future studies should examine whether or not a single-item indicator may be sufficient to capture this feeding aspect or if the remaining items should be kept. Similarly, in the FPSQ-S one item of the using food to calm construct (“I offer my child something to eat to make her/him feel better when she/he is hurt “) showed a standardised factor loading of 0.93 while the other five items’ loadings ranged from 0.65 to 0.87. Again, future work needs to examine if a single-item indicator may be sufficient. Interestingly, four items on the using (non-)food rewards construct had standardised factor loadings above 0.90. In this case, future studies need to examine if items from this construct should be removed for reasons of parsimony, especially if similarly high factor loadings are found for those four items again.

Factor-factor correlations of the FPSQ-M were all significant. The same was true for the equivalent four factors of the FPSQ-S, while four correlations involving the two additional factors did not reach significance. The positive correlations between using food as (non-)food rewards, using food to calm, persuasive and parent-led feeding (in the FPSQ-S) are in line with the positive correlations among the equivalent factors reported for the original FPSQ [22]. The findings for the FPSQ-M are the same, with the exception of a negative correlation between using food to calm and parent-led feeding. These findings indicate that even from an early age, these factors may cluster together representing a group of (non-) responsive feeding practices. As shown in Table 7, four out of six correlations were the same between the FPSQ original [22] and the FPSQ-M and 11 out of 15 correlations were the same between the FPSQ original and the FPSQ-S.

**Table 7:** Overview of factor-factor correlations in the FSPQ original, FPSQ-M and FPSQ-S

	Demand	Food to Calm	Persuasive Feeding	Parent-Led Feeding	Family Meal Environment
Demand (i.e. lower score) ≈ Structured Meal Timing	1				
Food to Calm ≈ Rewards for Behaviour	**Neg** Neg* Neg*	*1*			
Persuasive Feeding ≈ Persuasive Feeding	Pos* Pos* Pos*	Pos* Pos* Pos*	*1*		
Parent-Led Feeding ≈ Distrust	Pos* Pos* Pos*	Pos* Pos* Neg*	Pos* Pos* Pos*	*1*	
Family Meal Environment ≈ Family Meal Setting	**Pos** *Neg (*≈*0)*	Neg* *Neg*	Neg* Neg*	Neg* Neg*	*1*
Using (Non-) Food Rewards ≈ Rewards for Eating	**Pos** *Pos*	Pos* Pos*	Pos* Pos*	Pos* Pos*	Neg* *Pos*

Bold = original FPSQ [10], italics = FPSQ-S, underlined = FPSQ-M

During the development stage an inclusive approach was adopted and several items that showed issues with their distribution were kept for now while they would have been deleted if strict criteria for exclusion had been followed. Rather than directly discarding these items, future research will test them in diverse samples and contexts to examine their distribution again. Consequently, care needs to be taken particularly when interpreting the solid-feeding constructs rewards and using food to calm since they are based on data that showed high Kurtosis levels (often only three out of five responses were chosen). These data imply, possibly caused by a social desirability bias [35], that many parents report the ‘desired’ feeding behaviour at this early age. Only longitudinal research will be able to show the trajectories of feeding practices over time and the research team is currently working on further validating the measurement tool by utilising observational data and cognitive interviewing, which will help to explain whether these parental responses are true or artificial and caused either by social desirability, problems with the response scale or phrasing of the specific questions. Interestingly, items from the using food to calm construct showed a better distribution (specifically Kurtosis values) using the FPSQ-M compared to the FPSQ-S. Further research needs to clarify if this is an indication of age-appropriateness of the feeding construct or identify causes of these distributional differences. A second limitation to the study is the parent-reported nature of the measurement. Items of the FPSQ-M were phrased so that they are applicable to parents either breast- or bottle feeding and thus reducing bias related to parent feeding mode. Since the majority of respondents currently or previously breastfed their child, testing the FPSQ-M version in a predominantly bottle-fed sample is warranted. Additionally, while some participants contributed data to both questionnaire versions, this can be seen as a strength in that it allows for comparison of responses across the two versions and also ensures that for this measurement development stage parents with different feeding modes (only milk feeding, predominantly solid feeding, combination feeding) can respond to the questionnaires in a valid way. External reliability (e.g. test-retests) was not examined in the current study. Therefore, this and other psychometric testing is warranted in future studies. This also includes the expansion of the sample and context. The current sample lacked diversity with regards to socio-economic and feeding mode characteristics, as well as caregiver type and gender. Notably, Sample 1 included fathers, however the number was not large enough to look at this group separately during development or validation. We considered excluding fathers but finally decided to retain them since the goal for the future is to test the applicability of the FPSQ for infants and toddlers among fathers.

### Recommendations for future research

Further validation in diverse samples and contexts is indispensable. This may also include cognitive interviews with parents of infants and toddlers or comparisons of questionnaire data to direct observations of feeding interactions. Validation through examination of relationships with infant eating behaviour or weight is recommended as well. The same applies to external reliability testing, such as test-retest reliability. Finally, longitudinal assessment of feeding practices from milk to solid feeding in one sample will allow better understanding of the tracking of feeding practices across different developmental stages.

## Conclusion

In summary, although parental feeding practices have been identified as a risk factor for childhood overweight and obesity [14], the mechanisms and processes underlying such relationships are still uncertain. Mechanistic research is hindered by the lack of suitable tools to measure feeding practices across infancy and childhood. The milk and solid feeding versions of the FPSQ are newly developed and validated measures of feeding practices in early infancy. The development of the FPSQ-M and FPSQ-S allows for the measurement of relevant constructs founded in theory of authoritative feeding across infancy and childhood. This will facilitate research into the study of longitudinal processes and pathways of parental feeding and children’s eating/weight. The ability to measure the same underlying behaviours across childhood, starting at birth, will encourage tracking of feeding practices in order to ascertain how stable or malleable they are over time, how and why they may arise (e.g. from parent or child characteristics), how they may vary by feeding mode (breastfeeding, feeding from a bottle, solid foods), and their impact on child outcomes.

## Supplementary Information

**Additional file 1: **Tables showing excluded items and model fit. Description of data: **Table S1.** Excluded items (*n* = 7) – Milk-feeding version (FPSQ-M), **Table S2.** Excluded items (*n* = 22) – Solid-feeding version (FPSQ-S), **Table S3.** Model fit for the FPSQ-S across different CFA steps.

## Data Availability

The datasets used and/or analysed during the current study are available from the corresponding author on reasonable request.

## Abbreviations

FPSQ: Feeding practices and structure questionnaire
FPSQ-M: Feeding practices and structure questionnaire milk feeding version
FPSQ-S: Feeding practices and structure questionnaire solid feeding version
CFA: Confirmatory factor analysis
WLSMV: Weighted Least Squares Estimator
CFI: Comparative fit index
TLI: Tucker-Lewis index
RMSEA: Root mean-square error of approximation
