# Supplementary material for: The Feeding Practices and Structure Questionnaire: development and validation of age appropriate versions for infants and toddlers
Source: Int J Behav Nutr Phys Act. 2021 Jan 19;18:13. doi: 10.1186/s12966-021-01079-x (PMC7814443; doi:10.1186/s12966-021-01079-x)
Supplement: Supplementary file 1 — Additional file 1: Tables showing excluded items and model fit. Description of data: Table S1. Excluded items (n = 7) – Milk-feeding version (FPSQ-M), Table S2. Excluded items (n = 22) – Solid-feeding version (FPSQ-S), Table S3. Model fit for the FPSQ-S across different CFA steps. [file 12966_2021_1079_MOESM1_ESM.docx]

**Appendix**

Table 1: Excluded items (n=7) – Milk-feeding version (FPSQ-M)

| **Item** | **Theoretical construct** | **Why excluded?** |
| --- | --- | --- |
| I stop feeding when my baby shows that he is full (e.g. slowing pace of drinking, turning away, becoming unsettled) | Parent-led feeding | Distribution |
| I let my baby decide when he has finished feeding | Parent-led feeding | Distribution |
| I respond to my baby’s signs of hunger | Parent-led feeding | Distribution |
| I respond to my baby’s signs of fullness | Parent-led feeding | Distribution |
| I look for signs of fullness to decide when I stop feeding my baby (e.g. slowing pace, turning away, becoming unsettled) | Parent-led feeding | Distribution |
| I look for signs of hunger before I feed my baby (e.g. mouth opening, sucking, putting hand to mouth, seeking/rooting, turning head or squirming) | Parent-led feeding | Distribution |
| If my baby seems full, I stop trying to feed him | Persuasive feeding | Not loading in CFA |

Table 2: Excluded items (n=22) – Solid-feeding version (FPSQ-S)

| **Item** | **Theoretical construct** | **Why excluded?** |
| --- | --- | --- |
| I make my child sit down to eat. | Structure | Distribution |
| I let my child crawl or wander around while eating. | Structure | Not loading in CFA |
| I make sure my child eats in a highchair at the table. | Structure | Not loading in CFA |
| I let my child watch something on screen (TV, tablet, phone) while eating. | Structure | Not loading in CFA |
| My child plays with a toy when eating. | Structure | Not loading in CFA |
| I sit down with my child when they eat. | Structure | Distribution |
| I give my child milk or food whenever he seems to be hungry | Demand | DEM1 removed for parsimony, factor loading <0.6 |
| When deciding how much my child should eat, I rely on how hungry my child is. | Parent-led feeding | Not loading in CFA |
| When my child refuses food he usually eats, I still encourage him to eat. | Parent-led feeding | Overlapping with PERS 9 (When my child refuses food he usually eats, I encourage him to eat it) |
| When deciding how much my child should eat, I rely on the amount of food left in front of him/her | Parent-led feeding | PARENT3 removed for parsimony, factor loading <0.6 |
| I stop giving my child food when my child shows that they are full (for example: turning away, losing interest in food, getting distracted, spitting out or refusing more food). | Parent-led feeding | Distribution |
| I let my child decide when they have finished eating. | Parent-led feeding | Distribution |
| I respond to my child’s signs of hunger. | Parent-led feeding | Distribution |
| I respond to my child’s signs of fullness. | Parent-led feeding | Distribution |
| I look for signs of hunger before I give my child food (e.g. getting excited, leaning towards you, mouth opening). | Parent-led feeding | Distribution |
| I look for signs of fullness to decide when I stop giving my child food (e.g. turning away, losing interest in food, getting distracted, spitting out or refusing food). | Parent-led feeding | Distribution |
| I offer food to check if my child is hungry. | Persuasive feeding | Not loading in CFA |
| If my child seems full, I stop trying to give my child more food. | Persuasive feeding | Not loading in CFA |
| I let my child feed themselves as much as they want to. | Persuasive feeding | Not loading in CFA |
| I give my child extra food just to make sure he gets enough to eat | Persuasive feeding | PERS3 removed for parsimony, factor loading <0.6 |
| In order to get my child to behave themselves I promise my child something to eat. | Using (non-) food rewards | Distribution |
| I reward my child with something to eat when they are well behaved. | Using (non-) food rewards | Distribution |

Table 3: Model fit for the FPSQ-S across different CFA steps

| Model | Change | χ^2^ | df | RMSEA  (CI, p-value) | CFI | TLI |
| --- | --- | --- | --- | --- | --- | --- |
| 1 | Proposed model | 1679.399 | 371 | .076 (.072-.080, <.001) | .887 | .877 |
| 2 | Moved PERS5 to Parent-led factor – based on MI>59 | 1631.224 | 371 | .075 (.071-.078, <.001) | .891 | .881 |
| 3 | Remove PERS5 | 1500.409 | 344 | .074 (.070-.078, <.001) | .900 | .890 |
| 4 | Moved PERS11 to Parent-led factor – based on MI>24 | 1482.884 | 344 | .074 (.070-.077, <.001) | .901 | .892 |
| 5 | Remove PERS11 | 1398.719 | 318 | .075 (.071-.079, <.001) | .905 | .896 |
| 6 | Remove PARENT2 - factor loading <0.5 | 1311.122 | 293 | .075 (.071-.080, <.001) | .909 | .899 |
| 7 | Remove PERS6 - factor loading <0.5 | 1275.834 | 269 | .078 (.074-.083, <.001) | .906 | .896 |
| 8 | Remove PARENT1 (*When my child refuses food he usually eats, I still encourage him to eat*) because of high cross-loading with Persuasive feeding and great similarity to PERS9 (*When my child refuses food he usually eats, I encourage him to eat it*) | 883.362 | 246 | .065 (.061-.070, <.001) | .937 | .929 |
| 9 | Remove DEM1, PARENT3, PERS3 because factor loading <0.6, for parsimoniousness  🡪 Initial final model | 657.040 | 183 | .065 (.060-.071, <.001) | .949 | .942 |
| 9a | Model 9 in N=463 with extra items | 564.081 | 183 | .067 (.061-.073, <.001) | .953 | .947 |
| 10 | Added 2 factors – ‘Structure’ (8 items) & ‘Rewards’ (9 items) | 1942.010 | 650 | .066 (.062-.069, <.001) | .933 | .928 |
| 11 | Moved FME1 to Rewards factor – based on MI>213 | 1759.373 | 650 | .061 (.057-.064, <.001) | .942 | .938 |
| 12 | Deleted FME1 – factor loading <0.4 | 1688.866 | 614 | .061 (.058-.065, <.001) | .944 | .939 |
| 13 | Moved FME2 to Rewards factor – based on MI>196 | 1552.826 | 614 | .057 (.054-.061, <.001) | .951 | .947 |
| 14 | Deleted FME2 – factor loading <0.4 | 1434.327 | 579 | 0.56 (.053-.060, .002) | .955 | .951 |
| 15 | Deleted SIT3 – factor loading <0.4 | 1311.979 | 545 | 0.55 (.051-.059, .014) | .960 | .956 |
| 16 | Deleted SIT2 – factor loading <0.4  🡺 Final model | 1151.122 | 512 | .052 (.048-.056, .211) | .966 | .963 |

Abbreviations: Df= degrees of freedom, RMSEA = Root Mean-Square Error of Approximation, CFI = Comparative Fit Index, TLI = Tucker-Lewis Index, EFA = Exploratory Factor Analysis, MI = Modification Indices
